# Iguratimod Alleviates Myocardial Ischemia/Reperfusion Injury Through Inhibiting Inflammatory Response Induced by Cardiac Fibroblast Pyroptosis via COX2/NLRP3 Signaling Pathway

**DOI:** 10.3389/fcell.2021.746317

**Published:** 2021-10-25

**Authors:** Mian Zhang, Yi-shan Lei, Xiao-wen Meng, Hua-yue Liu, Lin-gui Li, Jun Zhang, Jia-xin Zhang, Wen-hui Tao, Ke Peng, Jun Lin, Fu-hai Ji

**Affiliations:** ^1^Department of Anesthesiology, First Affiliated Hospital of Soochow University, Suzhou, China; ^2^Institute of Anesthesiology, Soochow University, Suzhou, China; ^3^Department of Orthopedics, First Affiliated Hospital of Soochow University, Suzhou, China

**Keywords:** iguratimod, pyroptosis, inflammatory response, myocardial ischemia/reperfusion injury, cardiac fibroblasts, COX2, NLRP3

## Abstract

**Background:** NLRP3 inflammasome contributes a lot to sterile inflammatory response and pyroptosis in ischemia/reperfusion (I/R) injury. Cardiac fibroblasts (CFs) are regarded as semi-professional inflammatory cells and they exert an immunomodulatory role in heart. Iguratimod provides a protective role in several human diseases through exerting a powerful anti-inflammatory effect. However, it is still unclear whether iguratimod could alleviate myocardial I/R injury and whether inflammation triggered by NLRP3-related pyroptosis of CFs is involved in this process.

**Methods:** Transcriptomics analysis for GSE160516 dataset was conducted to explore the biological function of differentially expressed genes during myocardial I/R. *In vivo*, mice underwent ligation of left anterior descending coronary artery for 30 min followed by 24 h reperfusion. *In vitro*, primary CFs were subjected to hypoxia for 1 h followed by reoxygenation for 3 h (H/R). Iguratimod was used prior to I/R or H/R. Myocardial infarct area, serum level of cardiac troponin I (cTnI), pathology of myocardial tissue, cell viability, lactate dehydrogenase (LDH) release, and the expression levels of mRNA and protein for pyroptosis-related molecules were measured. Immunofluorescence was applied to determine the cellular localization of NLRP3 protein in cardiac tissue.

**Results:** During myocardial I/R, inflammatory response was found to be the most significantly enriched biological process, and nucleotide-binding oligomerization domain (NOD)-like receptor signaling was a crucial pathway in mediating cardiac inflammation. In our experiments, pretreatment with iguratimod significantly ameliorated I/R-induced myocardial injury and H/R-induced pyroptosis of CFs, as evidenced by reduced myocardial infarct area, serum cTnI level, and LDH release in supernatants, as well as improved pathology of cardiac tissue and cell viability. Immunofluorescence analysis showed that NLRP3 was mainly localized in CFs. Moreover, iguratimod inhibited the expression of pro-inflammatory cytokines and pyroptosis-related molecules, including NLRP3, cleaved caspase-1, and GSDMD-N.

**Conclusion:** Our results suggested that inflammatory response mediated by NOD-like receptor signaling is of vital importance in myocardial I/R injury. Iguratimod protected cardiomyocytes through reducing the cascade of inflammation in heart by inhibiting cardiac fibroblast pyroptosis via the COX2/NLRP3 signaling pathway.

## Introduction

Cardiovascular disease is a major cause of death and disability worldwide, among which ischemic heart disease represents a leading threat to human health (Ferraro et al., 2020). Despite that restoring blood perfusion rapidly is considered to be a chief treatment to limit the extent of myocardial infarction after cardiovascular events (Fröhlich et al., 2013; Hausenloy and Yellon, 2015), however, studies have proven that reperfusion triggered further cardiomyocyte death up to 50% of the overall infarcted location, which is defined as “myocardial ischemia/reperfusion (I/R) injury” (Yellon and Hausenloy, 2007). The pathophysiological mechanism of I/R injury is a rather complex process (Davidson et al., 2019; Heusch, 2020), of which inflammatory response is indicated to be a crucial factor in secondary myocardial damage (Eltzschig and Eckle, 2011; Frangogiannis, 2014). It is worthy of paying attention that cardiac fibroblasts (CFs), the most abundant non-cardiomyocytes in heart tissue, can sense danger signals released during cardiac injury or stress and participate in inducing the recruitment of immune cells, thus activating inflammatory response (Smolgovsky et al., 2021).

The nucleotide-binding oligomerization domain (NOD)-like receptor (NLR) is a kind of pattern recognition receptors, and its family members mainly participate in inflammasome formation, among which NLR with a pyrin domain 3 (NLRP3) inflammasome is the most extensively studied. It has been well established that NLRP3 inflammasome, an important signaling sensor platform containing NLRP3, apoptosis-associated speck-like protein containing a CARD (ASC) and caspase-1, is essential for pyroptosis (Nagata and Tanaka, 2017; Bedoui et al., 2020). Pyroptosis, a newly discovered process of pro-inflammatory programmed cell death, is featured by pore formation in the plasma membrane and extracellular release of pro-inflammatory factors. Inflammatory caspases, including caspase-1, caspase-4, and caspase-5, cleave gasdermin family member gasdermin D (GSDMD) into C-terminal and N-terminal, with the latter translocating to the inner leaflet of plasma membrane and mediating cell pyroptosis (Shi et al., 2015; Nagata and Tanaka, 2017). Previous researches have indicated the critical role of pyroptosis in cardiovascular diseases and concluded that excessive pyroptosis may have a detrimental effect on cardiomyocytes (Zhou et al., 2018; An et al., 2019; Shi et al., 2021). Results from animal experiments demonstrated that inhibition of caspase-1 or knockdown of ASC and NLRP3 could prevent myocardium injury (Kawaguchi et al., 2011; Sandanger et al., 2013; Audia et al., 2018). In addition, it was reported that NLRP3 inflammasome induced by myocardial I/R was mainly expressed in non-cardiomyocytes, such as fibroblasts, endothelial cells, and infiltrated leukocytes within the myocardium (Kawaguchi et al., 2011; Sandanger et al., 2013; Liu et al., 2014). Therefore, interventions in NLRP3-mediated pyroptosis of CFs may reduce inflammatory counterparts and further exert myocardial protection.

Iguratimod, an emerging antirheumatic drug, has been characterized with selectively inhibitory role in the activity and induction of COX2 (Tanaka et al., 1992, 1995). Recently, investigators studied the therapeutic effect of iguratimod on multiple disease models (Jiang et al., 2018; Li et al., 2018, 2019), and the results suggested that iguratimod improved these diseases by exerting strong anti-inflammatory effect. Moreover, it was reported that iguratimod could inhibit NLRP3 inflammasome (Hou et al., 2019). Results from another study showed that COX2 positively regulated the expression of NLRP3 and IL-1β in macrophages (Hua et al., 2015). Nonetheless, to the best of our knowledge, the biological effect of iguratimod on COX2/NLRP3 signaling pathway in myocardial I/R model still remains elusive.

Transcriptomics studies gene transcription and function through analyzing all RNA from tissues or cells. Recently, transcriptomics has been widely applied in human disease-related researches and it can reveal underlying molecular mechanisms comprehensively (Singhania et al., 2018; Montaner et al., 2020). GSE160516 dataset stores expression data from mouse heart tissue treated with I/R, which can show the gene expression alteration associated with the development of cardiac injury. Therefore, we carried out bioinformatics analysis for GSE160516 dataset to identify differentially expressed genes (DEGs) and their biological function.

Collectively, the present study aimed to provide theoretical basis for a comprehensive understanding of myocardial I/R injury from a perspective of transcriptomics and to investigate the role and underlying mechanism of iguratimod on I/R-induced cardiomyocyte injury. We hypothesized that iguratimod pretreatment could reduce inflammatory response in cardiac tissue and protect the myocardium, and these effects were mediated by downregulation of CF pyroptosis via suppressing the COX2/NLRP3 signaling pathway.

## Materials and Methods

### Microarray Data and Identification of Differentially Expressed Genes

The myocardial I/R microarray dataset was downloaded from the Gene Expression Omnibus (GEO) database^1^ : GSE160516. There were four mouse heart tissues in the sham surgery group (sham), I/R for 6 h group (IR6h), I/R for 24 h group (IR24h), and I/R for 72 h group (IR72h), respectively. The gene expression data (GSE160516) were detected by the Affymetrix Clariom S Mouse Array platform (GPL23038). Boxplots and principal component analysis (PCA) were used to visualize the expression profiles of the four groups. GEO2R was utilized to identify DEGs in IR24h samples compared to sham samples, setting the thresholds of Benjamini-Hochberg (BH) method adjusted *p*-value <0.05 and | log_2_ (fold change)| ≥ 1 (| log_2_FC| ≥ 1) as the cutoff line. The visualization of the results was performed using the R software (version 4.0.4) and OmicShare tools^2^, presented as volcano plot, bar charts, and heatmap.

### Gene Ontology and Pathway Enrichment Analysis

**FIGURE 1:**
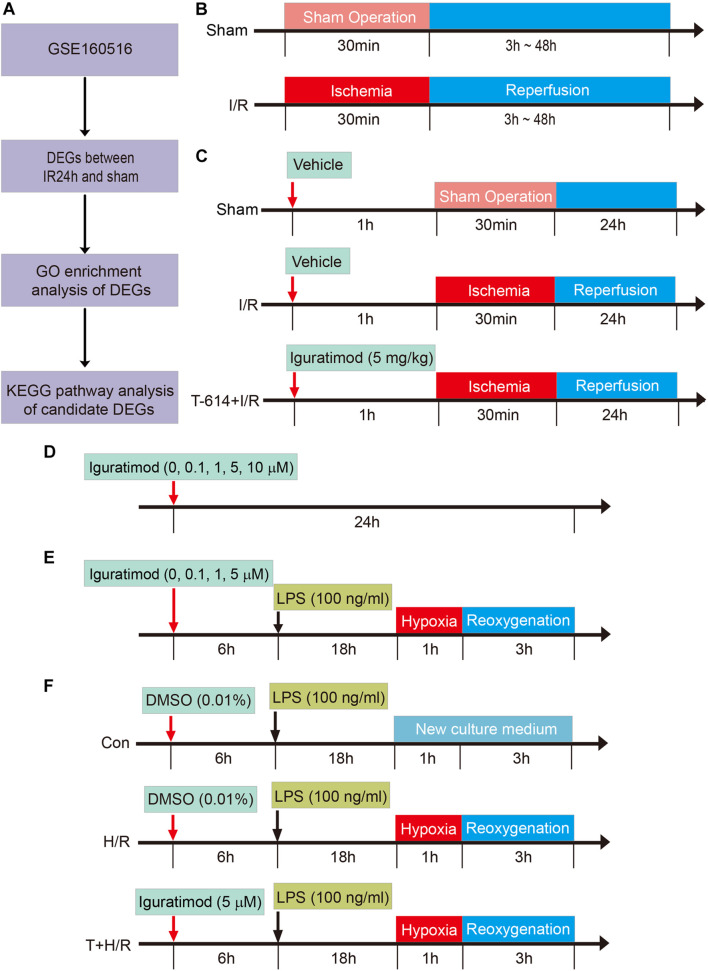
Schematic diagram of the experimental design and major process in the present study. **(A)** Flow chart of processing data in GSE160516 dataset. **(B)** Experiment design 1 was performed to demonstrate the time course of changes in mRNA expression of COX2 and NLRP3 after myocardial reperfusion. **(C)** Experiment design 2 was conducted to investigate the therapeutic effect of Iguratimod pretreatment on myocardial injury induced by I/R and the underlying mechanism. **(D,E)** Experiment design 3 was carried out to explore the optimal concentration of iguratimod for mitigating H/R injury of primary cardiac fibroblasts. **(F)** Experiment design 4 assessed the implications of iguratimod on pyroptosis of cardiac fibroblasts in *vitro* H/R model. I/R, ischemia/reperfusion; H/R, hypoxia/reoxygenation.

Gene Ontology (GO) analysis is mainly applied to carry out the biological function enrichment of the proteins encoded by DEGs. The Metascape online database^3^ was used to perform GO analysis, which contains three different terms for the molecular function, biological process, and cellular component categories (Zhou et al., 2019). Subsequently, the pathway enrichment analysis for the DEGs that were included in the most statistically significant GO term was conducted using the Kyoto Encyclopedia of Genes and Genomes (KEGG) pathways online tool^4^. The enrichment results of the GO term and KEGG pathway analysis were visualized by GraphPad Prism 8 software (GraphPad Software, San Diego, CA, United States). The process of analyzing data in GSE160516 dataset is shown in Figure 1A.

### Experiment Design *in vivo* and *in vitro*

#### Experiment Design 1: Changes of COX2 and NLRP3 Expression in Heart Tissue of Myocardial Ischemia/Reperfusion Mice Model

In order to determine the changes of COX2 and NLRP3 mRNA expression in heart tissue at various reperfusion time points, mice were randomly allocated into the following five groups: sham group (Sham), reperfusion 3 h group [I/R(3h)], reperfusion 6 h group [I/R(6h)], reperfusion 24 h group [I/R(24h)], and reperfusion 48 h group [I/R(48h)]. All mice were sacrificed at an indicated time point after myocardial reperfusion and the heart tissue below ligated position was collected for real-time quantitative PCR (RT-qPCR) to access mRNA expression levels of COX2 and NLRP3 (Figure 1B). The experimental data were statistically analyzed, and thus, we chose 24 h as the optimal reperfusion time.

#### Experiment Design 2: Therapeutic Effect of Iguratimod on Ischemia/Reperfusion-Induced Cardiomyocyte Injury and the Potential Molecular Mechanisms

Adult male C57BL/6 mice were divided into the following three groups: Sham group, I/R group, and Iguratimod pretreatment (T-614) + I/R group. Mice in T-614 + I/R group received intraperitoneal injection of iguratimod (Cat no. HY-17009; MedChemExpress, Monmouth Junction, NJ, United States) at a dose of 5 mg/kg 1 h before myocardial ischemia. Mice in another two groups were administrated with equal volume solvent of 5% DMSO intraperitoneally (Figure 1C).

After myocardial reperfusion for 24 h, mice were sacrificed for following experiments. The myocardial infarct and ischemia area were assessed by Evans blue and 2,3,5-triphenyl tetrazolium chloride (TTC) double staining. The concentration of cardiac troponin I (cTnI) in serum was measured by enzyme-linked immunosorbent assay (ELISA). Hematoxylin and eosin (HE) staining was used to observe the pathological morphology of cardiac tissue. The expression of myeloperoxidase (MPO) in heart was detected by immunohistochemistry (IHC) staining. The mRNA levels of COX2, NLRP3, and some inflammatory factors including IL-1β, IL-6, IL-18, and TNF-α were measured by RT-qPCR. Western blotting was adopted to detect relative expression levels of COX2, NLRP3, caspase-1, ASC, cleaved caspase-1, GSDMD-N, and IL-1β. Additionally, the cellular localization of NLRP3 in cardiac tissue was determined by immunofluorescence (IF) staining.

#### Experiment Design 3: The Optimal Concentration of Iguratimod for *in vitro* Experiments in Primary Cardiac Fibroblasts

Cardiac fibroblasts were incubated with iguratimod of different concentrations (0, 0.1, 1, 5, 10 μM, respectively) for 24 h at normal culture condition, to find the optimal concentration with no cytotoxicity (Figure 1D). CFs were then pretreated with iguratimod (0, 0.1, 1, and 5 μM, respectively) for 6 h, followed by LPS (100 ng/ml) incubation for 18 h, and were then subjected to hypoxia for 1 h and reoxygenation for 3 h (H/R) (Figure 1E). Cell counting kit-8 (CCK-8) and lactate dehydrogenase (LDH) release were applied to select the optimal concentration of iguratimod *in vitro* H/R model.

#### Experiment Design 4: Effect of Iguratimod on H/R-Induced Pyroptosis of Cardiac Fibroblasts and Expression of Pro-inflammatory Factors

Murine primary CFs were divided into three groups: Con group, H/R group, and iguratimod pretreatment (T) + H/R group. Cells in the Con group were incubated with 100 ng/ml of LPS for 18 h followed by normal medium culture for 4 h. CFs in the H/R group were treated with LPS for 18 h before H/R. The T + H/R group was pretreated with iguratimod of 5 μM for 6 h, followed by the same procedure of the H/R group. At the end of reoxygenation, cells of all groups were harvested for accessing the mRNA expressions of COX2, NLRP3, IL-1β, IL-6, IL-18, and TNF-α, and the protein levels of COX2, NLRP3, caspase-1, ASC, cleaved caspase-1, GSDMD-N, and IL-1β (Figure 1F).

### Animals

Healthy adult male C57BL/6 mice (20–25 g weight) were purchased from Cavens Biogle (Suzhou) Model Animal Research Co., Ltd. (Animal license No. SCXK Jiang-su 2018-0002). All of the animals were housed in a specific room with controlled environmental condition (a normal 12 h light-dark cycle and constant temperature and humidity) and were fed with commercial food and water. The animal experimental procedures were carried out in line with the guide for the care and use of laboratory animals [National Institutes of Health (NIH), Bethesda, MD, United States] and also passed the review of the Institutional Animal Care and Use Committee of Soochow University (Suzhou, China).

### Establishing Myocardial Ischemia/Reperfusion Model *in vivo*

Mice were anesthetized by intraperitoneal administration of 1% pentobarbital sodium at a dose of 50 mg/kg. They were ventilated with a rodent respirator (Shenzhen Ruiwode Life Technology Co., Ltd., China) after tracheal intubation. The thorax was opened along the left fourth intercostal space to expose the heart. A 6-0 suture thread with needle was tied into a slipknot to ligate the left anterior descending coronary artery (LAD) at 3 mm under the extension line of the lower edge of the left auricle of the heart. The mark of successful ischemia was cyanosis in left ventricular myocardium wall or ST-segment elevation on the electrocardiograph. Following 30-min ischemia, the ligation was removed to allow reperfusion of the occluded coronary artery and then the incision was sutured carefully. All the mice were returned to their cages after recovering from anesthesia and were given free access to food and water.

### Isolation and Culture of Primary Cardiac Fibroblasts

Murine primary CFs were isolated from hearts of neonatal C57BL/6 mice (aged 1–3 days) with a differential adhesion method as previously described (Wu et al., 2016). In brief, hearts from neonatal mice were harvested promptly and chopped into small pieces under aseptic condition. To gain suspension of CFs, cardiac tissue was digested with 0.25% trypsin (Cat no. C0201; Beyotime, China) at 37°C followed by dissociation in collagenase type 2 (0.5 mg/ml; Cat no. V900892; Sigma, St. Louis, MO, United States). The cell suspension was filtered through a 200-μm cell strainer and then centrifuged at 1000 × *g* for 5 min. DMEM/F12 medium (Cat no. C11330500BT, Gibco; Invitrogen, Carlsbad, CA, United States) containing 10% fetal bovine serum (FBS), and 1% penicillin/streptomycin was added into the precipitation to resuspend the cells. After pre-culture for 70 min at 37°C, CFs were obtained by differential wall-adherence durations. Subsequently, the purity of primary CFs was confirmed by immunocytochemistry with an anti-vimentin monoclonal antibody (1:100, Cat no. 5741; CST, Danvers, MA, United States). CFs of passage 2, which were grown to 80% confluence in culture dishes, were used for *vitro* experiments.

### Hypoxia/Reoxygenation Model *in vitro*

Primary CFs were treated with glucose-free DMEM (Cat no. PM150271; Procell, China) and pre-incubated in chamber filled with mixed gas (95% N_2_ and 5% CO_2_) at 37°C for 1 h. Subsequently, cells were cultured in complete medium with DMEM/F12 containing 10% FBS under normal atmosphere (95% air, 5% CO_2_) at 37°C for 3 h.

### Evans Blue and 2,3,5-Triphenyl Tetrazolium Chloride Double-Staining

At an indicated time point after reperfusion, mice were anesthetized and the LAD was ligated again *in situ*; 1% Evans blue solution (Cat no. E2129; Sigma) was injected into the inferior vena cava, after which hearts were resected, washed with cold saline, and frozen at –80°C for 15 min. Frozen hearts were then transversely sectioned into slices with 2-mm thickness and incubated in phosphate buffer solution (pH = 7.4) containing 1% TTC (Cat no. T8877; Sigma) at 37°C for 30 min. Subsequently, the slices were fixed in 4% paraformaldehyde fix solution (Cat no. BL539A; Biosharp, China). An Image J software (National Institutes of Health) was used to calculate the infarct area (INF) and area at risk (AAR). The infarcted size of the heart was regarded as the percentage of INF/AAR.

### Enzyme-Linked Immunosorbent Assay

The serum level of cTnI was determined as a biomarker of myocardial damage. In the present study, murine blood sample was drawn from the inferior vena cava and collected in tubes for serum isolation. The concentration of cTnI was measured by an ELISA kit (Cat no. CTNI-1-HS; Life Diagnostics, West Chester, PA, United States) according to the manufacturer’s instructions.

### Hematoxylin and Eosin Staining

Hematoxylin and eosin staining was used to observe the histological structure of heart tissue. Briefly, paraformaldehyde-fixed and paraffin-embedded hearts were cut transversely into 5-μm-thick sections. After dewaxing with dimethylbenzene and dehydrating with graded ethanol series, the heart slices were stained with hematoxylin for 5 min and then alcohol- soluble eosin for 30 s. Finally, these sections were observed under a light microscope following sealing with neutral gum.

### Immunohistochemistry Staining

For IHC, paraffin-embedded heart tissue sections were firstly deparaffinized and rehydrated. Subsequently, antigen was retrieved by heating samples in Tris-based buffer (pH = 9.0) to boil for 15 min. After three times of washing, the tissue slides were blocked with 5% BSA for 1 h and then incubated with primary antibody of myeloperoxidase (1:500, Cat no. ab208670; Abcam, Cambridge, MA, United States) at 4°C overnight in a humidified chamber. The following day, slices were incubated with appropriate HRP-conjugated secondary antibody (1:200, Cat no. ab6721; Abcam) for 1 h at 37°C. Positive staining could be visualized after using a DAB kit. At last, the representative images were further quantified for calculating the expression of MPO by using Image J software.

### Western Blotting

After reperfusion for 24 h, cardiac tissues were harvested and homogenized in frozen RIPA lysis buffer (Cat no. P0013B; Beyotime) mixed with phenylmethylsulfonyl fluoride (Cat no. ST506; Beyotime). Then, the homogenate was centrifuged at 14,800 × *g* for 30 min, and the supernatant extracts were sucked out. The protein concentration was determined by BCA method. Equal amounts of total protein were loaded in each well and then separated by 10% sodium dodecyl sulfate–polyacrylamide gel electrophoresis. The protein was then transferred to a polyvinylidene difluoride (PVDF) membrane. Subsequently, the PVDF membrane was sealed with 5% non-fat milk in 1× Tris-buffered saline tween (TBST) buffer for 2 h followed by incubation with primary antibodies (Table 1) at 4°C overnight. After three times of washing, the PVDF membrane was incubated with corresponding horseradish peroxidase-labeled anti-rabbit or anti-mouse secondary antibodies (1:4000, Cat no. CW0102S, Cat no. CW0103S; CoWin Biosciences, China) at room temperature for 2 h. The immunoblot bands were visualized by means of an electrochemiluminescence kit (Tanon, China) and the intensity of the bands was measured using the Image J software. The final semi-quantitative results were presented as the ratios of targeted molecular intensity relative to that of β-tubulin.

**TABLE 1:** Antibodies applied in Western blotting.

Antibodies	Cat. no. and company	Dilution ratio
COX2	ab14191, Abcam	1:1000
NLRP3	15101, CST	1:500
Caspase-1	CY10200, Abways	1:500
ASC	67824, CST	1:500
IL-1β	abs131179, absin	1:500
Cleaved caspase-1	89332, CST	1:500
GSDMD	ab219800, Abcam	1:500
β-Tubulin	abs137976, absin	1:20000

### RNA Extraction and Real-Time Quantitative PCR

Total RNA of heart tissue was extracted using Trizol reagent (Cat no. 15596026; Thermo Fisher Scientific, Carlsbad, CA, United States) according to manufacturer’s instructions. Quality and purity of RNA were identified by UV spectrophotometry (NanoDrop 2000; Thermo Fisher Scientific) at 260 and 280 nm, respectively; 1 μg of total RNA was used to synthesize cDNA. The qPCR reaction mixture contained 5 μl of SYBR qPCR SuperMix (Cat no. E096-01A; Novoprotein, China), 1 μl of forward and reverse primers, 1 μl of cDNA, and 2 μl of RNase-free water. The qPCR cycling was programmed for 40 cycles followed by the construction of a melting curve. Using a single predominant peak as quality control, qPCR was applied to estimate the transcript levels of selected genes: COX2, NLRP3, IL-1β, IL-18, TNF-α, and IL-6 with β-tubulin as normalization of mRNA expression. Data were analyzed by using the 2^–ΔΔCt^ method. Primer sequences of mice mRNA (Table 2) were synthesized by Shanghai Sangon Biotech Co., Ltd.

**TABLE 2:** Primer sequences used in quantitative polymerase chain reaction in this study.

Primer name	Sequence (5′ → 3′)	
COX-2	Forward	ATTCCAAACCAGCAGACTCATA
	Reverse	CTTGAGTTTGAAGTGGTAACCG
NLRP3	Forward	CCAGACACTCATGTTGCCTGTTC
	Reverse	GAGGCTCCGGTTGGTGCTTA
IL-1β	Forward	CACTACAGGCTCCGAGATGAACAAC
	Reverse	TGTCGTTGCTTGGTTCTCCTTGTAC
IL-18	Forward	AGACCTGGAATCAGACAACTTT
	Reverse	TCAGTCATATCCTCGAACACAG
IL-6	Forward	ACAACCACGGCCTTCCCTACTT
	Reverse	CACGATTTCCCAGAGAACATGTG
TNF-α	Forward	AAGCCTGTAGCCCACGTCGTA
	Reverse	GGCACCACTAGTTGGTTGTCTTTG
β-Tubulin	Forward	GGGAGGTGATAAGCGATGAA
	Reverse	AGGGACATACTTGCCACCTG

### Immunofluorescence Staining

Hearts were resected and fixed in 4% paraformaldehyde for at least 1 day to prepare for immunofluorescence analysis. Thereafter, the specimens were soaked in gradient sucrose solutions in PBS to dehydrate and were processed into frozen sections. Representative sections from the ischemic region were permeabilized in 0.5% Triton X-100 for 15 min and sealed with 5% BSA for 1 h. Afterwards, the sections were incubated with the following primary antibodies at 4°C for 12 h: anti-COX2 (1:100, Cat no. ab15191; Abcam), anti-NLRP3 (1:100, Cat no. NBP2-12446; Novus, Littleton, CO, United States), anti-Vimentin (1:100, Cat no. 5741; CST) and anti-Smooth Muscle Actin (1:100, Cat no. sc-53142; Santa Cruz Biotechnology, Santa Cruz, CA, United States). Primary antibodies were detected by Alexa Fluor 488-conjugated goat anti-rabbit (1:500, Cat no. ab150077; Abcam), Alexa Fluor 568-conjugated goat anti-rabbit (1:500, Cat no. ab175471; Abcam), or Alexa Fluor 488-conjugated goat anti-mouse (1:500, Cat no. ab150113; Abcam) secondary antibodies, respectively, for 2 h at room temperature. Nuclei were identified with its marker of 4′,6-diamidino-2-phenylindole (DAPI) (Cat no. 62248; Thermo Fisher Scientific). Finally, the sections were observed with a fluorescence microscope.

### Cell Counting Kit-8

Cell proliferation and cytotoxicity assay were measured using Cell Counting Kit-8 (CCK-8 kit) (Cat no. CK04; Dojindo, Japan). In brief, primary CFs were seeded in 96-well plates at a density of 10,000 cells/well and were pre-cultured for 24 h in a humid condition (5% CO_2_, 37°C). After corresponding processing, the supernatant in each well was supplemented with 10 μl of CCK-8 solution and then the mixture was incubated at 37°C for 3 h. A microplate reader was used to detect the absorbance at 450 nm.

### Lactate Dehydrogenase Release in Supernatant

Primary CFs were seeded in 96-well plates at a density of 15,000 cells/well and were pre-incubated for 24 h. To measure LDH release, 60 μl of supernatant in each well of the plate was transferred to a new plate. According to manufacturer’s instructions, the homogeneous reaction solution containing 10 μl of lactic acid, 10 μl of diaphorase, and 10 μl of iodonitrotetrazolium (INT) was added into the supernatant and then this mixture was incubated at room temperature for 30 min in the dark. At last, the absorbance of each well was measured at 490 nm with a microplate reader.

### Statistical Analysis

Data from the GSE160516 dataset were processed with the R software, online bioinformatic databases, and tools as mentioned before. The GraphPad Prism 8 software was applied to analyze experimental data. Continuous variables were presented as mean ± standard deviation (SD). Data between two groups with normal distribution were assessed using the two-sided unpaired Student *t*-test, and the differences among multiple groups were evaluated using ordinary one-way or two-way analysis of variance (ANOVA) followed by Tukey’s multiple comparisons test. In both cases, a *p*-value of less than 0.05 was considered statistically meaningful.

## Results

### Inflammatory Response Mediated by Nucleotide-Binding Oligomerization Domain-Like Receptor Signaling Pathway Plays an Important Role in Myocardial Ischemia/Reperfusion Injury

The raw data in GSE160516 dataset were normalized and standardized (Supplementary Figure 1). The distribution of all samples was shown in the form of a two-dimensional scatter diagram with PCA (Supplementary Figure 2). The PCA plot also indicated that samples within each group were similar to each other and there were significant discriminations among four groups.

**FIGURE 2:**
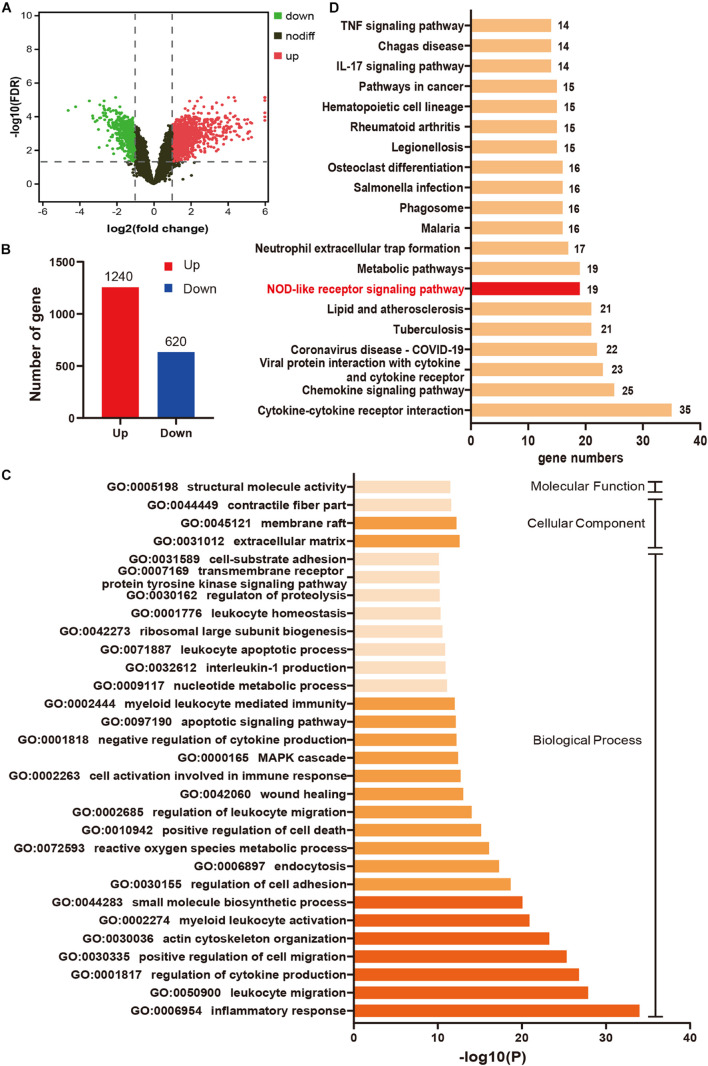
Inflammatory response mediated by NOD-like receptor signaling pathway plays an important role in myocardial I/R injury. **(A)** Volcano plot for DEGs between IR24h samples and sham samples. Red dots and green dots respectively represent up-regulated and down-regulated genes in IR24h samples compared to sham samples (adjusted *p*-value < 0.05 and | log_2_FC| ≥ 1). Black dots indicate the genes without significant expression difference between the two groups (adjusted *p*-value ≥ 0.05 or | log_2_FC| <1). **(B)** The number of up-regulated and down-regulated genes in IR24h samples compared to sham samples. **(C)** Heatmap of GO analysis enriched terms colored by *p*-value. GO functional enrichment analysis classified the DEGs into three functional groups: molecular function group, biological process group, and cellular component group. **(D)** KEGG pathway analysis for the DEGs enriched in GO:0006954. NOD-like receptor signaling pathway is marked with red color. DEGs, differentially expressed genes; log_2_FC, log_2_ (fold change); GO, Gene ontology; KEGG, Kyoto Encyclopedia of Genes and Genomes.

A total of 1860 DEGs (1240 upregulated genes and 620 downregulated genes) were identified in IR24h samples compared to sham samples (Figures 2A,B). The top 20 significant DEGs are listed in Supplementary Table 1. As shown in Figure 2C, the top 30 terms of GO enrichment analysis for DEGs were demonstrated as bar graphs, among which there were 26 items in the biological process group, three items in cellular component group, and one item in the molecular function group (more details shown in Supplementary Table 2). The result demonstrated that these DEGs were mainly enriched in leukocyte, cytokine, and cell migration-related processes. Among these GO terms, inflammatory response (GO:0006954) was the most significant enrichment process, with 176 DEGs included (gene list shown in Supplementary Table 3). The enriched top 20 KEGG pathways for these 176 DEGs were demonstrated in Figure 2D (details shown in Supplementary Table 4), among which there were 19 genes enriched in NOD-like receptor signaling pathway.

### COX2 and Molecules Associated With Pyroptosis Were Both Upregulated After Myocardial Ischemia/Reperfusion

**FIGURE 3:**
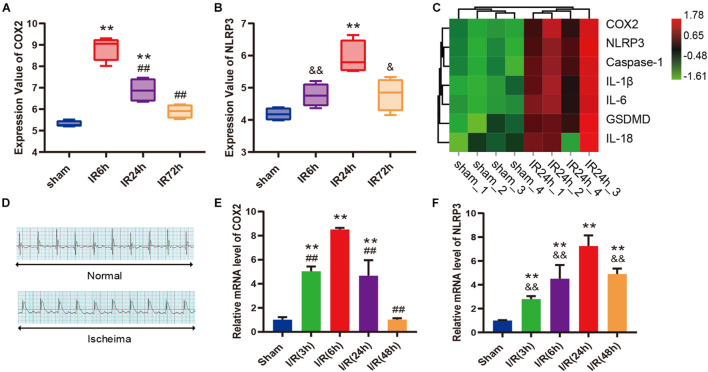
COX2 and molecules associated with pyroptosis were both up-regulated after myocardial I/R. **(A,B)** Transcriptional expression values of COX2 and NLRP3 in sham samples and I/R samples of GSE160516 dataset. *n* = 4. **(C)** Expression heatmap of COX2, NLRP3, caspase-1, IL-1β, IL-6, GSDMD, and IL-18 in sham samples and IR24h samples. Each column represents a sample and each row represents expression profile of a specific gene. The color scale demonstrates the relative gene expression level in certain grid: green and red indicate the low and high expression values of the gene, respectively. **(D)** Representative electrocardiograph before and after ligation of LAD *in vivo* model of myocardial I/R. **(E,F)** Changes in mRNA expression levels of COX2 and NLRP3 after myocardial reperfusion. *n* = 5. Data are expressed as the mean ± SD; **p < 0.01 versus sham group or Sham group; ^ ##^*p* < 0.01 versus IR6h group or I/R(6h) group; ^&^*p* < 0.05, ^&&^*p* < 0.01 versus IR24h group or I/R(24h) group. I/R, ischemia/reperfusion; LAD, left anterior descending coronary artery.

As shown in box plots (Figures 3A,B), the transcriptional expression of COX2 and NLRP3 in cardiac tissue changed dynamically with the reperfusion time, showing a trend of increasing first and then decreasing. To be specific, COX2 expression reached the peak at 6 h after myocardial reperfusion, and the expression peak of NLRP3 was at 24 h. The heatmap demonstrated that the expression levels of COX2, NLRP3, Caspase-1, IL-1β, IL-6, GSDMD, and IL-18 gene were all upregulated in IR24h samples (Figure 3C). Subsequently, we successfully established the mice model of myocardial I/R, which was identified by an elevation of ST segment on electrocardiograph (Figure 3D). qPCR results further confirmed similar expression trends of COX2 and NLRP3 in left ventricular myocardium after reperfusion (Figures 3E,F). Based on these findings, 24 h after myocardial reperfusion was selected as the optimal time point in subsequent *in vivo* experiments.

### Iguratimod Ameliorated Myocardial Injury and Infiltration of Inflammatory Cells After Ischemia/Reperfusion

**FIGURE 4:**
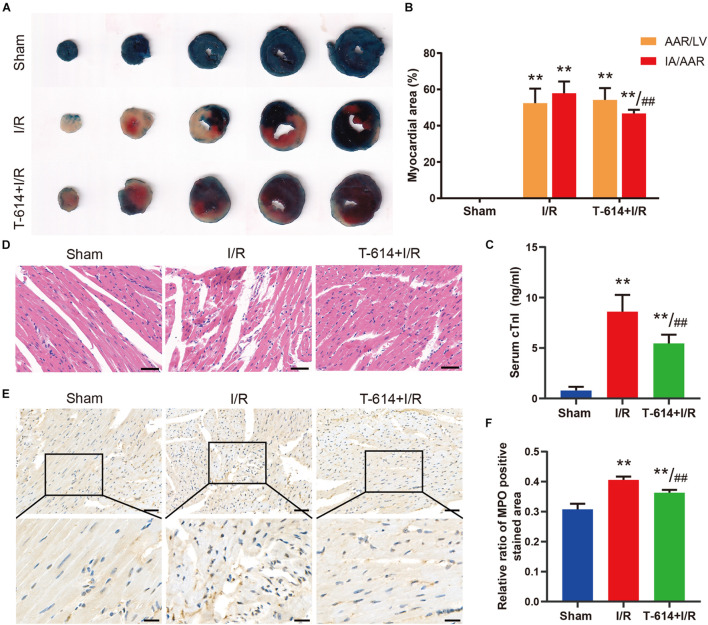
Iguratimod ameliorated myocardial injury and infiltration of inflammatory cells after I/R. **(A)** Representative images of Evans blue and TTC double-stained heart sections. **(B)** Comparison of AAR/LV ratio and IA/AAR ratio among the three groups based on the results of **A**. *n* = 5. **(C)** The level of cTnI in serum determined by ELISA. *n* = 5. **(D)** Representative HE staining pictures of hearts from mice in three groups. Scale bar = 50 μm. **(E)** Representative immunohistochemistry staining images of MPO in cardiac tissue. Scale bar of the first row = 50 μm and scale bar of the second row = 20 μm. **(F)** Comparison of the MPO positive stained area among the three groups. *n* = 4. Data are expressed as mean ± SD; **p < 0.01 versus Sham group; ^ ##^*p* < 0.01 versus I/R group. I/R, ischemia/reperfusion; AAR, area at risk; LV, left ventricle; IA, infarct area; cTnI, cardiac troponin I; MPO, myeloperoxidase.

As shown in the Evans blue and TTC double stained heart sections, the AAR/LV ratio and IA/AAR ratio both increased significantly in I/R group and T-614 + I/R group compared with the Sham group. While there was no difference of AAR/LV ratio between the I/R group and T-614 + I/R group, the ratio of IA/AAR decreased significantly in the latter group, indicating that iguratimod pretreatment limited the infarct size after myocardial I/R (Figures 4A,B). The concentration of cTnI in serum was elevated after I/R and was reduced by iguratimod pretreatment (Figure 4C). HE staining showed that mice in the Sham group had clear and intact heart tissue structure, and the cardiomyocytes were arranged in order (Figure 4D). Nevertheless, the hearts from the I/R group were characterized with unclear tissue structure, swollen cardiomyocytes, disrupted myofibrils, and inflammatory cell infiltration. In the T-614 + I/R group, iguratimod ameliorated the above-mentioned morphological damage, with milder myofibril fracture and inflammatory cell infiltration. The result of IHC staining demonstrated that the MPO-positive stained area in the T-614 +I/R group was smaller than that in the I/R group, which indicated less neutrophil infiltration and mitigated inflammatory response (Figures 4E,F).

### The Alleviated Myocardial Ischemia/Reperfusion Injury in Iguratimod Pre-treated Mice Was Associated With Inhibition of Pyroptosis and Inflammatory Response Mediated by COX2/NLRP3 Signaling Pathway

**FIGURE 5:**
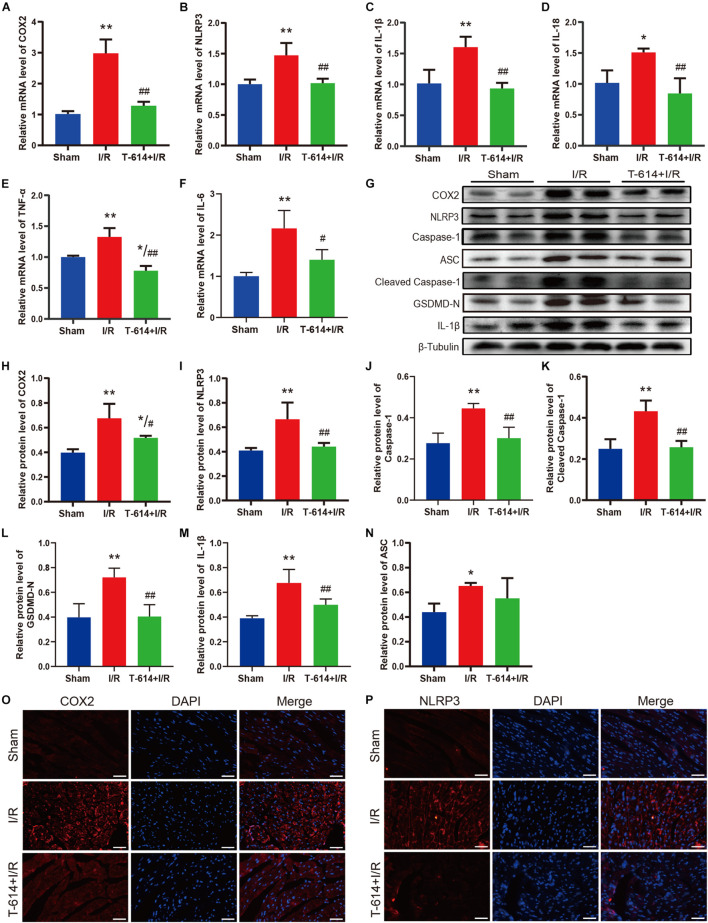
Pyroptosis and inflammatory response mediated by COX2/NLRP3 signaling pathway were suppressed by iguratimod. **(A–F)** Relative mRNA expression levels of COX2, NLRP3, IL-1β, IL-18, IL-6, and TNF-α among Sham, I/R and T-614 + I/R group. *n* = 4. **(G)** Representative Western blotting luminogram of COX2, NLRP3, caspase-1, ASC, cleaved caspase-1, GSDMD-N, and IL-1β of the three groups. **(H–N)** Protein semiquantification standardized by β-Tubulin. *n* = 5. **(O,P)** Representative immunofluorescence staining pictures of COX2 and NLRP3 in left ventricular myocardium from hearts of different mice. Scale bar = 50 μm. Data are expressed as mean ± SD;*p < 0.05, **p < 0.01 versus Sham group; ^ #^*p* < 0.05, ^##^*p* < 0.01 versus I/R group. I/R, ischemia/reperfusion.

As shown in Figures 5A–F, cardiac tissue from I/R group exhibited obviously increased mRNA levels of COX2, NLRP3, IL-1β, IL-18, IL-6, and TNF-α in comparison to the Sham group. When mice were pre-treated with iguratimod before being subjected to myocardial ischemia, the mRNA levels of the above-mentioned molecules all reduced significantly. Moreover, results of the Western blotting analysis demonstrated that iguratimod pretreatment rendered a remarkable decline in the protein expression of COX2, NLRP3, caspase-1, cleaved caspase-1, GSDMD-N, and IL-1β, which were induced by I/R dramatically (Figures 5G–M). However, in the protein level of ASC, there was a lack of significant difference between the I/R group and T-614 + I/R group, though showing a trend of decrease after iguratimod pretreatment (Figure 5N). In addition, pictures of immunofluorescence staining showed lower expression of COX2 and NLRP3 in left the ventricular myocardium tissue within the T-614 + I/R group, when compared with the I/R group (Figures 5O,P). Taken together, these findings indicated that iguratimod attenuated pyroptosis and inflammatory response triggered by I/R through the inhibition of COX2/NLRP3 pathway.

### Iguratimod Attenuated Cell Injury of Primary Cardiac Fibroblasts Exposed to H/R Condition

**FIGURE 6:**
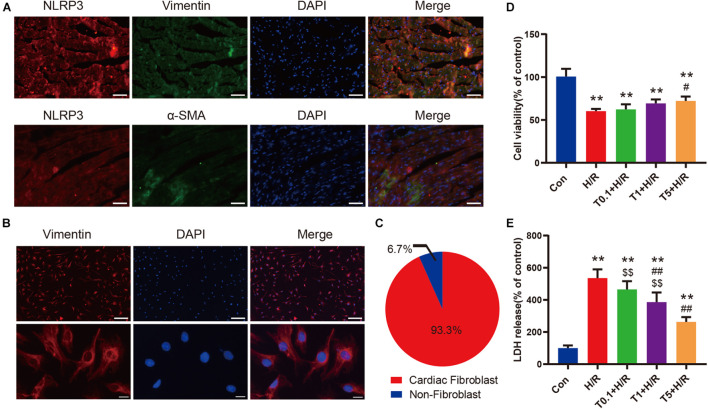
Iguratimod attenuated cell injury of primary CFs exposed to H/R condition. **(A)** Double-immunofluorescence staining with antibodies against NLRP3 (red) and vimentin (green) or α-SMA (green) for left ventricular myocardium tissue. Scale bar = 50 μm. **(B)** Representative images of primary CFs isolated from hearts of neonatal mice. Scale bar of the first row = 50 μm and scale bar of the second row = 5 μm. **(C)** The purity of primary CFs in the present study. **(D)** Cell viability of primary CFs in Con, H/R and Iguratimod (0.1, 1, 5 μM, respectively) pretreatment + H/R group. *n* = 5. **(E)** Percentage of LDH release in cell culture supernatants among the five groups. *n* = 6. Data are expressed as mean ± SD; **p < 0.01 versus Con group; ^ #^*p* < 0.05, ^##^*p* < 0.01 versus H/R group; ^$$^*p* < 0.01 versus T5 + H/R group. CFs, cardiac fibroblasts; H/R, hypoxia/reoxygenation; α-SMA, α-smooth muscle actin; LDH: lactate dehydrogenase.

The results of double immunofluorescence staining showed that there was more colocalization of NLRP3-positive cells with vimentin-positive cells in the left ventricular myocardium tissue after myocardial I/R (Figure 6A). Considering that vimentin was a special cytoskeleton protein of fibroblasts, we concluded that upregulated NLRP3 was expressed mainly in CFs instead of cardiomyocytes. Thereafter, primary CFs were isolated from mice aged 1–3 days to directly explore the effect of iguratimod on CFs exposed to H/R. The representative images of primary CFs, which were identified by immunofluorescence staining of vimentin, are presented in Figure 6B. The purity of primary CFs used in *in vitro* experiments was up to 93.3% (Figure 6C). As shown in Supplementary Figures 3A,B, iguratimod had no detrimental effect on CFs when its concentration ranged from 0 to 5 μM, as indicated by normal cell viability and low LDH release in the supernatants. Additionally, 100 ng/ml LPS did not cause injury to CFs (Supplementary Figures 4A,B). Therefore, LPS was used in simulated I/R experiments *in vitro* for priming of NLRP3 inflammasome. It can be seen from Figures 6D,E that H/R induced severe injury of CFs, as evidenced by decreased cell viability and elevated LDH release. Interestingly, compared with the H/R group, pretreatment with iguratimod increased cell viability only at a high concentration of 5 μM (Figure 6D), while it significantly decreased LDH release at concentration of both 1 and 5 μM (Figure 6E). Therefore, 5 μM of iguratimod was applied for the following experiments *in vitro*.

### Iguratimod Ameliorated Pyroptosis and the Expression of Pro-inflammatory Cytokines of Cardiac Fibroblasts Subjected to H/R Through Inhibiting the COX2/NLRP3 Signaling Pathway

**FIGURE 7:**
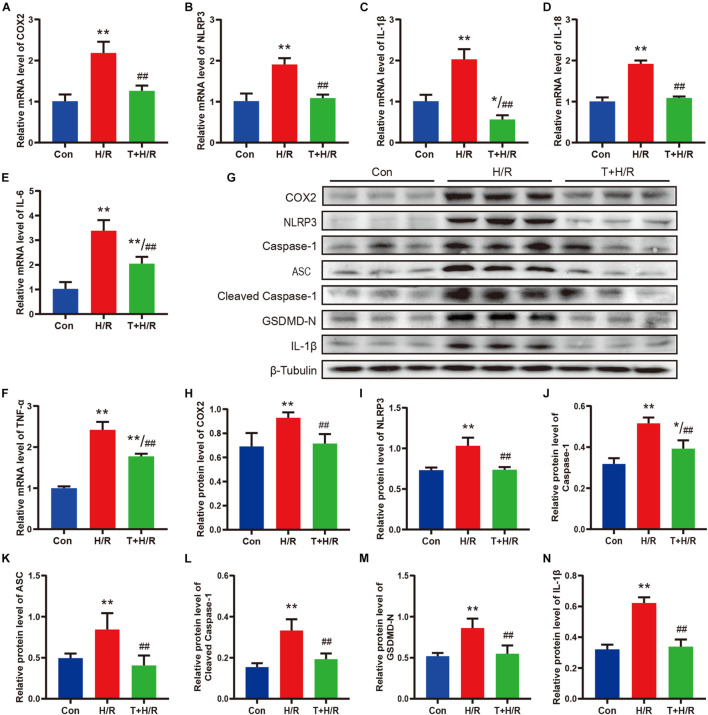
Iguratimod ameliorated pyroptosis and the expression of pro-inflammatory cytokines of CFs subjected to H/R through inhibiting the COX2/NLRP3 signaling pathway. **(A–F)** Relative mRNA expression levels of COX2, NLRP3, IL-1β, IL-18, IL-6, and TNF-α among Con, H/R and T + H/R group. *n* = 4. **(G)** Representative Western blotting luminogram of COX2, NLRP3, caspase-1, ASC, cleaved caspase-1, GSDMD-N, and IL-1β of the three groups. **(H–N)** Protein semiquantification standardized by β-Tubulin. *n* = 5. Data are expressed as mean ± SD; *p < 0.05, **p < 0.01 versus Con group; ^##^*p* < 0.01 versus H/R group. CFs, cardiac fibroblasts; H/R, hypoxia/reoxygenation.

As shown in Figures 7A–F, H/R greatly elevated the mRNA levels of COX2, NLRP3, IL-1β, IL-18, IL-6, and TNF-α in primary CFs. However, the transcriptional levels of these genes were all downregulated by iguratimod of 5 μM. Similarly, the protein expression levels of COX2, NLRP3, caspase-1, ASC, cleaved caspase-1, GSDMD-N, and IL-1β were all significantly decreased in the T + H/R group in comparison with the H/R group (Figures 7G–N). Conclusively, the above results suggested that H/R greatly promoted pyroptosis of primary CFs, whereas pretreatment with Iguratimod remarkably mitigated pyroptosis and subsequent release of initial inflammatory mediators, as indicated by reduced expression of cell pyroptosis biomarker, GSDMD-N, and pro-inflammatory cytokines.

## Discussion

**FIGURE 8:**
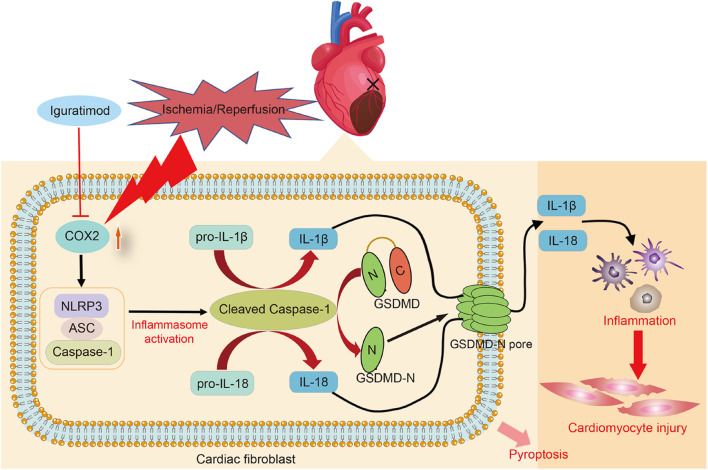
Schematic diagram of this study: the primary mechanism of Iguratimod pretreatment ameliorating myocardial ischemia/reperfusion injury. Specifically, the expression of COX2 in cardiac fibroblasts was elevated after cardiac I/R, leading to the upregulation and activation of NLRP3 inflammasome. Activated caspase-1 cut GSDMD into GSDMD-N, which further executed pyroptosis. Additionally, pro-IL-1β and pro-IL-18 were also cleaved by activated caspase-1 and subsequently their mature forms were released into extracellular region and triggered amounts of inflammatory cells infiltration in the cardiac tissue, causing inflammatory reperfusion injury. Iguratimod reduced inflammatory cytokines secretion and cardiac inflammation response through inhibiting pyroptosis of cardiac fibroblasts via acting on COX2/NLRP3 signaling pathway, eventually improving myocardial I/R injury. I/R, ischemia/reperfusion.

The present study found that DEGs mainly participated in inflammatory response during myocardial I/R, and NOD-like receptor signaling pathway played a crucial role in mediating inflammation of cardiac tissue. Pretreatment with iguratimod significantly ameliorated excessive cardiac inflammation and cardiomyocytes injury induced by I/R. Moreover, iguratimod decreased the expression of COX2, NLRP3 inflammasome, and pro-inflammatory factors in both *in vivo* and *in vitro* experiments, indicating that the cardioprotective effect of iguratimod was partly mediated through inhibiting CF pyroptosis-triggered inflammatory response via COX2/NLRP3 signaling pathway (Figure 8).

Myocardial I/R often causes irreversible changes in cardiac structure and function, as well as injury and death of cardiomyocytes, which involve in many complex pathological processes, including inflammatory response (Zuurbier et al., 2019), oxidative stress (Bugger and Pfeil, 2020), intracellular calcium overload (Wang et al., 2020), etc. Prior studies demonstrated that aseptic inflammation induced by myocardial ischemia was one of the important factors increasing myocardial infarct size (Mezzaroma et al., 2011; Frangogiannis, 2014). Consistently, the present study showed that DEGs during myocardial I/R were mainly involved in immune and inflammation-related biological processes, such as inflammatory response, leukocyte migration, regulation of cytokine production, positive regulation of cell migration, etc. I/R alters the balance between anti-inflammatory and pro-inflammatory cytokines, and the latter exacerbate inflammatory response and ischemic injury (Toldo et al., 2018). Specifically, subpopulations of blood-derived cells are delivered to the ischemic area with the help of pro-inflammatory molecules, and then they closely adhere to viable cardiomyocytes, release destructive enzymes into the environment, produce ROS and cause cytotoxic effect, thus extending I/R injury (Frangogiannis, 2014; Gunata and Parlakpinar, 2021).

Pyroptosis is one of the highly pro-inflammatory lytic forms of programmed cell death, which is featured by permeabilization of the plasma membrane, cell swelling, and release of pro-inflammatory factors (Shi et al., 2021). It is well accepted that cleaved caspase-1, the functional performer of activated NLRP3 inflammasome, cleaves GSDMD into C-terminal and N-terminal, and the latter mediates cell pyroptosis (Nagata and Tanaka, 2017; Bedoui et al., 2020). Moreover, cleaved caspase-1 also cuts inactive pro-IL-1β and pro-IL-18 to mature IL-1β and IL-18. Therefore, NLRP3 inflammasome, GSDMD, IL-1β, and IL-18 are all recognized as pyroptosis-related molecules (Zhang et al., 2020; Mo et al., 2021; Nie et al., 2021). These two interleukins, which are released to extracellular space through GSDMD-N pore in the cell membrane, are the upstream of the inflammation and can act as important signal amplifiers to induce the secretion of additional cytokines, chemokines, and adhesion molecules, thus triggering the inflammatory cascade and eventually causing cell damage (Venkatachalam et al., 2009; Eltzschig and Eckle, 2011; Kim et al., 2013). Similarly, we found that NLRP3-mediated pyroptosis signaling was differentially expressed and enriched in inflammatory response process during myocardial I/R through bioinformatics analysis.

In *in vivo* experiments, the up-regulation of pyroptosis-related molecules was observed in the left ventricular myocardium tissue, indicating that I/R triggered cell pyroptosis. Double immunofluorescence staining of cardiac tissue sections showed that I/R-induced NLRP3 had more co-localization with vimentin, the specific cytoskeleton of fibroblasts. This result was consistent with previous studies, which concluded that inflammasome-related genes trigged by myocardial I/R, including NLRP3, IL-1β, and IL-18, were expressed mainly in cardiac fibroblasts and microvascular endothelial cells, instead of cardiomyocytes (Kawaguchi et al., 2011; Sandanger et al., 2013). Activation of NLRP3 inflammasome in non-cardiomyocytes did not directly lead to the loss of contractile structure but caused cardiac dysfunction indirectly by amplifying the inflammatory response through IL-1β and IL-18 (Kawaguchi et al., 2011). According to a recent study, fibroblasts can sense the danger signals released during cardiac injury or stress and participate in inducing the recruitment of immune cells to activate and maintain the inflammatory response (Smolgovsky et al., 2021). In view of the increasing focus on the immune role of CFs, we further investigated the effect of H/R on CFs in *in vitro* experiments, and the results demonstrated that H/R induced pyroptosis of primary CFs, confirmed by rupture of cell membrane (as assessed by LDH release) and enhanced expression of pyroptosis-related molecules. Taken together, NLRP3-mediated pyroptosis of CFs may cause injury to myocardium through triggering excessive inflammation.

Iguratimod, a novel drug commonly used in antirheumatic diseases, had been reported to possess therapeutic effect on colitis (Jiang et al., 2018), multiple sclerosis (Li et al., 2018), tumor, and neurodegenerative diseases in animal models (Li et al., 2019) due to its powerful anti-inflammatory ability. Likewise, our research was the first to explore the effect of iguratimod on myocardial I/R injury, and the results demonstrated that iguratimod reduced the myocardial infarct size, cTnI level in serum, and pathological changes of cardiac tissue, indicating its cardioprotective role. Moreover, iguratimod also suppressed the inflammatory response in heart, confirmed by decreased neutrophil infiltration and expression of inflammatory cytokines. Recently, investigators had revealed the inhibitory effect of iguratimod on NLRP3 inflammasome in mice model of acute pancreatitis (Hou et al., 2019). Similarly, in our study, the molecular expressions of NLRP3, caspase-1, cleaved caspase-1, GSDMD-N, and IL-1β were all decreased after iguratimod pretreatment in *in vivo* and *in vitro* experiments. Although the expression level of ASC protein in cardiac tissue was not dramatically reduced by iguratimod, it remarkably fell off in CFs exposed to H/R in *in vitro* experiments. This result suggested the specific effect of iguratimod on inflammasome in non-cardiomyocytes. Therefore, the cardioprotective role of iguratimod was closely associated with suppressing NLRP3 inflammasome-mediated pyroptosis of CFs.

It has been well-established that up-regulation of COX2 is clearly related to the pathogenesis of many inflammatory processes, and therapeutic treatment targeting COX2 is commonly recommended for inflammatory diseases (Bagi et al., 2006; Redondo et al., 2011; Díaz-Muñoz et al., 2012). Hua et al. (2015) reported for the first time that silencing COX2 gene or using COX2 inhibitors both reduced the expression and activation of NLRP3 inflammasome and downstream cytokines and ameliorated caspase-1-dependent cell apoptosis. Subsequently, results from other studies also supported the theory that COX2 was involved in the activation of NLRP3 inflammasome (Daniels et al., 2016; Lu et al., 2017; Hou et al., 2019). In this study, data from microarray dataset and *in vivo* experiment demonstrated that the transcriptional expression peak of COX2 gene was a little earlier than that of NLRP3 gene after myocardial I/R. Additionally, we found consistent changes in the expression of COX2 and NLRP3 with and without iguratimod pretreatment, indicating the potential regulatory role of COX2 for NLRP3 inflammasome in myocardial I/R. Evidence from previous literature has demonstrated that iguratimod is a highly selective inhibitor of COX2, with inhibition of its gene expression and enzyme activity (Tanaka et al., 1995). In pancreatitis model, the down-regulated expression of COX2 and NLRP3 was observed after preconditioning with iguratimod (Hou et al., 2019). Likewise, our experiments showed that I/R-induced COX2 expression was significantly decreased by iguratimod pretreatment, suggesting that the potential target of iguratimod in alleviating myocardial I/R injury could be COX2.

To sum up, the present study revealed the important role of inflammatory response in I/R-induced myocardial injury and the cardioprotective effect of iguratimod, which mitigated inflammation-mediated myocardium injury through inhibiting pyroptosis of cardiac fibroblasts via down-regulation of COX2/NLRP3 signaling pathway. These findings might provide important insights into the potential therapeutic role of iguratimod in myocardial I/R injury.

However, there were also limitations in the present study. Firstly, the secretion levels of inflammatory factors in cellular culture supernatants, such as IL-1β and IL-18, were not detected with ELISA, which could directly illustrate the inflammatory microenvironment generated by pyroptosis of CFs and better demonstrate the initial immune role of CFs in the inflammatory cascade after I/R. Secondly, we did not transfect the CFs with overexpression plasmid of COX2. Therefore, there was insufficient evidence to further elucidate that iguratimod exerted an inhibitory role on NLRP3 through acting on COX2. Finally, although neonatal primary cells have been widely used in the study of cardiovascular disease, there may be difference between neonatal and adult primary cells. In the future, further researches are required to clarify other underlying mechanisms in the cardioprotective effect of iguratimod.

## Data Availability Statement

The dataset presented in this study can be found in Gene Expression Omnibus (GEO) database (http://www.ncbi.nlm.nih.gov/geo). The name of the repository and accession number is: GSE160516. The original contributions presented in the study are included in the article/Supplementary Material, further inquiries can be directed to the corresponding authors.

## Ethics Statement

The animal study was reviewed and approved by the Institutional Animal Care and Use Committee of Soochow University (Suzhou, China).

## Author Contributions

MZ, X-WM, and F-HJ contributed to the conception and design of the study. MZ and L-GL processed GSE160516 dataset and drew the plots. MZ conducted the experiments and drafted the manuscript. Y-SL, JL, and F-HJ critically reviewed and offered significant modifications to the manuscript. J-XZ, JZ, KP, H-YL, and W-HT cooperatively helped collect the data and perform statistical analysis. All the authors approved the final version of the manuscript.

## Conflict of Interest

The authors declare that the research was conducted in the absence of any commercial or financial relationships that could be construed as a potential conflict of interest.

## Publisher’s Note

All claims expressed in this article are solely those of the authors and do not necessarily represent those of their affiliated organizations, or those of the publisher, the editors and the reviewers. Any product that may be evaluated in this article, or claim that may be made by its manufacturer, is not guaranteed or endorsed by the publisher.

## Footnotes

1
http://www.ncbi.nlm.nih.gov/geo

2
https://www.omicshare.com/tools/

3
http://metascape.org

4
https://www.kegg.jp/kegg/
