# Supplementary material for: Iguratimod Alleviates Myocardial Ischemia/Reperfusion Injury Through Inhibiting Inflammatory Response Induced by Cardiac Fibroblast Pyroptosis via COX2/NLRP3 Signaling Pathway
Source: Front Cell Dev Biol. 2021 Oct 25;9:746317. doi: 10.3389/fcell.2021.746317 (PMC8573346; doi:10.3389/fcell.2021.746317)
Supplement: Supplementary file 1 [file Data_Sheet_1.docx]

Supplementary Material

# Supplementary Figures

##
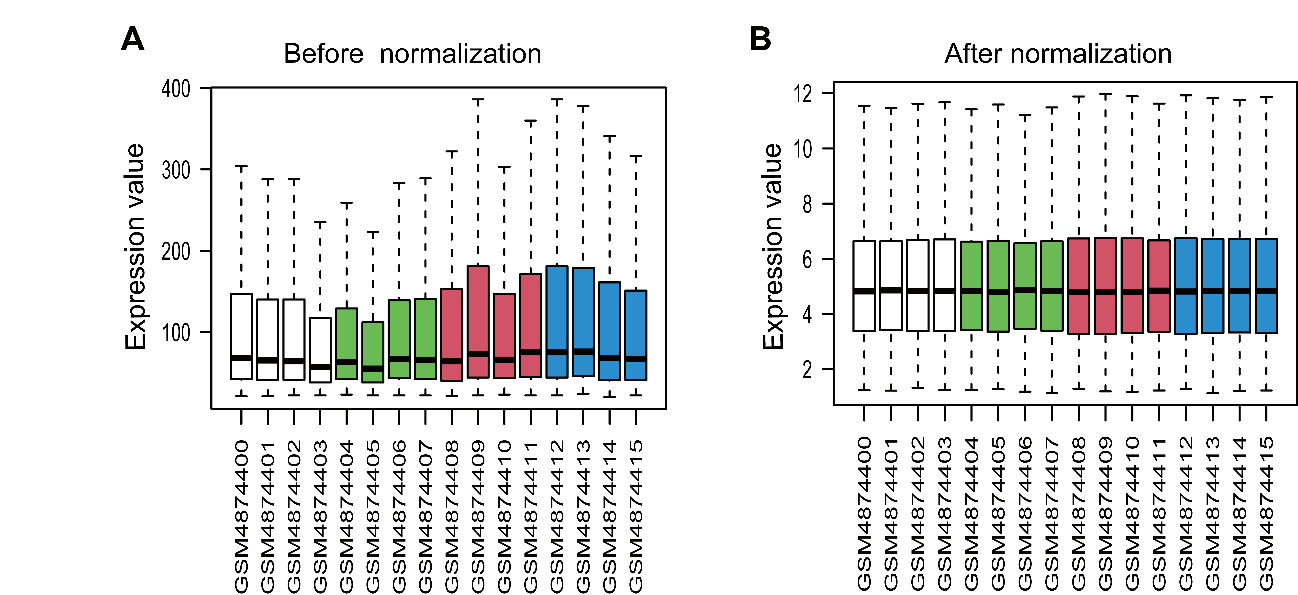
Supplementary Figure 1

**Supplementary Figure 1.** Boxplots for GSE160516 dataset (**A**) prior to and (**B**) after application of normalization. White boxes represent sham samples. Green boxes represent IR6h samples. Pink and blue boxes represent IR24h samples and IR72h samples, respectively.

## Supplementary Figure 2


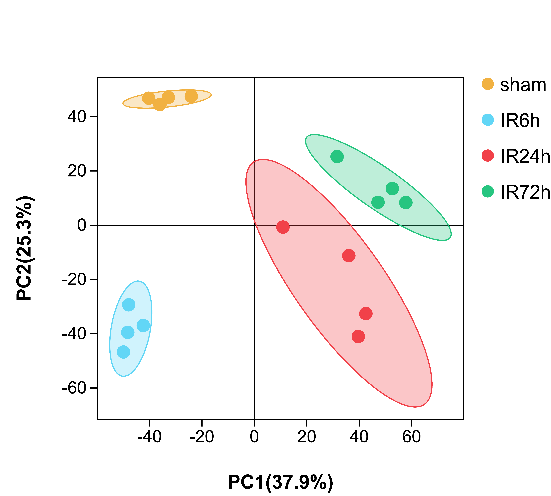


**Supplementary Figure 2.** PCA for mRNA expression of the sixteen samples in GSE160516 dataset. PCA: principal component analysis.

## Supplementary Figure 3


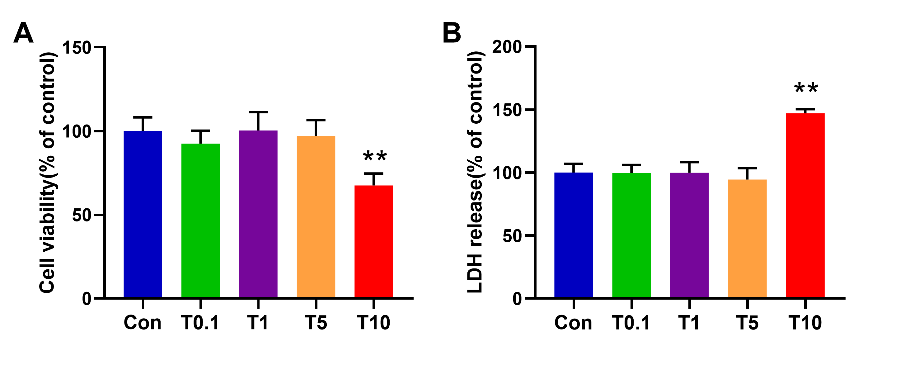


**Supplementary Figure 3.** The effect of Iguratimod at different concentrations on primary CFs cultured in normal condition. (**A**) Cell viability of primary CFs, which were incubated with Iguratimod of different concentrations, was assessed by CCK-8 assay. n=5. (**B**) Percentage of LDH release in cell culture supernatants. n=5. Data are expressed as the mean ± SD; ^**^*P* < 0.01 versus Con group. CFs: cardiac fibroblasts; CCK-8: cell counting kit-8; LDH: lactate dehydrogenase.

## Supplementary Figure 4


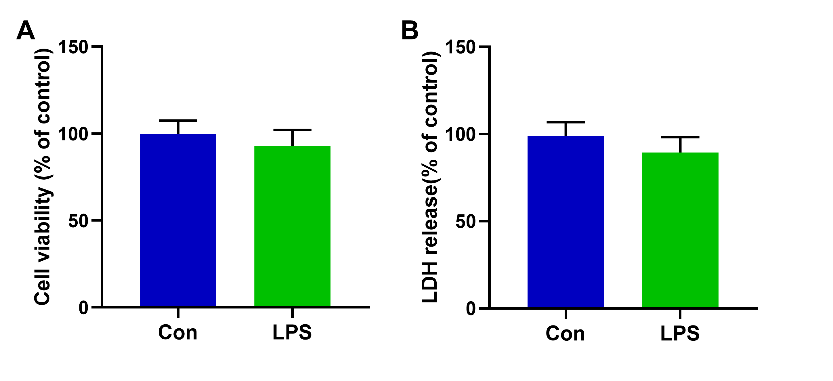


**Supplementary Figure 4.** The effect of LPS (100 ng/ml) on primary CFs cultured in normal condition. (**A**) Cell viability of primary CFs, which were incubated with or without LPS (100 ng/ml), was assessed by CCK-8 assay. n=5. (**B**) Percentage of LDH release in cell culture supernatants. n=5. Primary CFs were incubated with or without LPS (100 ng/ml). Data are expressed as the mean ± SD. CFs: cardiac fibroblasts; CCK-8: cell counting kit-8; LDH: lactate dehydrogenase.

# Supplementary Tables

## Supplementary Table 1

**Supplementary Table 1.** 1860 DEGs were identified between sham samples and IR24h samples in GSE160516 dataset. Among these DEGs, 1240 genes were up-regulated and 620 genes were down-regulated (here only the top 20 significant DEGs were listed).

| Gene symbol | *P*-value | adj. *P*-value | log_2_FC | Regulated | Chromosome |
| --- | --- | --- | --- | --- | --- |
| ANKRD2 | 1.03E-09 | 7.59E-06 | 8.21 | up-regulated | chr19 |
| VLDLR | 1.96E-09 | 7.59E-06 | -2.05 | down-regulated | chr19 |
| SERPINB1A | 1.18E-09 | 7.59E-06 | 4.04 | up-regulated | chr13 |
| NMRK2 | 2.10E-09 | 7.59E-06 | 6.53 | up-regulated | chr10 |
| TIMP1 | 5.37E-10 | 7.59E-06 | 8.59 | up-regulated | chrX |
| FGL2 | 8.43E-10 | 7.59E-06 | 2.56 | up-regulated | chr5 |
| TNC | 1.97E-09 | 7.59E-06 | 7.46 | up-regulated | chr4 |
| UCK2 | 1.97E-09 | 7.59E-06 | 4.34 | up-regulated | chr1 |
| PRG4 | 3.54E-09 | 1.14E-05 | 6.3 | up-regulated | chr1 |
| PKD212 | 4.78E-09 | 1.2E-05 | -3.47 | down-regulated | chr18 |
| Gm6682 | 5.82E-09 | 1.24E-05 | 1.94 | up-regulated | chr12 |
| FHL1 | 5.00E-09 | 1.2E-05 | 4.4 | up-regulated | chrX |
| LGALS1 | 6.03E-09 | 1.24E-05 | 2.36 | up-regulated | chr15 |
| TUBB2A | 8.03E-09 | 1.55E-05 | 3.39 | up-regulated | chr13 |
| ALDH1A2 | 1.18E-08 | 2.06E-05 | 3.7 | up-regulated | chr9 |
| ASB15 | 1.22E-08 | 2.06E-05 | -2.9 | down-regulated | chr6 |
| TCAP | 1.56E-08 | 2.48E-05 | -2.7 | down-regulated | chr11 |
| GLIPR2 | 1.63E-08 | 2.48E-05 | 2.23 | up-regulated | chr4 |
| VCAN | 1.90E-08 | 2.692E-05 | 2.71 | up-regulated | chr13 |
| TUBB2B | 2.05E-08 | 2.69E-05 | 3.16 | up-regulated | chr13 |

## Supplementary Table 2

**Supplementary Table 2.** GO enrichment analysis of DEGs (here only the top 30 significant GO terms were listed).

| GO term | Category | Description | -log10(P) | Count | % |
| --- | --- | --- | --- | --- | --- |
| GO:0006954 | GO biological process | Inflammatory response | 33.989 | 176 | 23.53 |
| GO:0050900 | GO biological process | Leukocyte migration | 27.891 | 105 | 28.85 |
| GO:0001817 | GO biological process | Regulation of cytokine production | 26.799 | 160 | 21.77 |
| GO:0030335 | GO biological process | Positive regulation of cell migration | 25.33 | 133 | 23.37 |
| GO:0030036 | GO biological process | Actin cytoskeleton organization | 23.259 | 150 | 20.92 |
| GO:0002274 | GO biological process | Myeloid leukocyte activation | 20.899 | 73 | 30.29 |
| GO:0044283 | GO biological process | Small molecule biosynthetic process | 20.057 | 140 | 20.11 |
| GO:0030155 | GO biological process | Regulation of cell adhesion | 18.677 | 142 | 19.32 |
| GO:0006897 | GO biological process | Endocytosis | 17.298 | 145 | 18.49 |
| GO:0072593 | GO biological process | Reactive oxygen species metabolic process | 16.083 | 75 | 24.92 |
| GO:0010942 | GO biological process | Positive regulation of cell death | 15.166 | 131 | 18.25 |
| GO:0002685 | GO biological process | Regulation of leukocyte migration | 14.015 | 58 | 26.73 |
| GO:0042060 | GO biological process | Wound healing | 13.031 | 84 | 20.79 |
| GO:0002263 | GO biological process | Cell activation involved in immune response | 12.7 | 69 | 22.70 |
| GO:0031012 | GO cellular component | Extracellular matrix | 12.588 | 98 | 19.03 |
| GO:0000165 | GO biological process | MAPK cascade | 12.407 | 132 | 16.77 |
| GO:0045121 | GO cellular component | Membrane raft | 12.226 | 79 | 20.73 |
| GO:0001818 | GO biological process | Negative regulation of cytokine production | 12.219 | 63 | 23.33 |
| GO:0097190 | GO biological process | Apoptotic signaling pathway | 12.129 | 109 | 17.90 |
| GO:0002444 | GO biological process | Myeloid leukocyte mediated immunity | 12.022 | 36 | 33.33 |
| GO:0044449 | GO cellular component | Contractile fiber part | 11.585 | 52 | 25.24 |
| GO:0005198 | GO molecular function | Structural molecule activity | 11.489 | 107 | 17.63 |
| GO:0009117 | GO biological process | Nucleotide metabolic process | 11.093 | 103 | 17.64 |
| GO:0032612 | GO biological process | Interleukin-1 production | 10.922 | 35 | 31.53 |
| GO:0071887 | GO biological process | Leukocyte apoptotic process | 10.860 | 40 | 28.57 |
| GO:0042273 | GO biological process | Ribosomal large subunit biogenesis | 10.536 | 27 | 37.50 |
| GO:0001776 | GO biological process | Leukocyte homeostasis | 10.317 | 35 | 30.17 |
| GO:0030162 | GO biological process | Regulation of proteolysis | 10.224 | 120 | 16.15 |
| GO:0007169 | GO biological process | Transmembrane receptor protein tyrosine kinase signaling pathway | 10.206 | 102 | 17.11 |
| GO:0031589 | GO biological process | Cell-substrate adhesion | 10.120 | 71 | 19.83 |

## Supplementary Table 3

**Supplementary Table 3.** 176 DEGs were enriched in inflammatory response process (GO:0006954).

| GO term | Enriched genes |  |
| --- | --- | --- |
| GO:0006954 | | Acp5, Adam8, Adcy7, Adora1, Adora2b, Agtr1a, Aif1, Alox5ap, Bst1, Btk, C1qa, C3, C3ar1, C5ar1, Casp1, Casp4, Cd14, Cd24a, Cd44, Cd59a, Cd68, Chil3, Clu, Cxcr2, Ccr1, Ccr2, Ccr5, Csf1r, Ctla2a, Ctsc, Ctss, Cybb, Cd55, Dpep1, Ednra, Ephx2, F3, Fcer1g, Fcgr1, Fcgr2b, Fcgr3, Fn1, Fpr2, Fpr1, Lilrb4a, Gpx1, Cxcl1, Gstp1, Hck, Hgf, Hmox1, Hp, Ier3, Cxcl10, Igf1, Il17ra, Il1b, Il1r1, Il1r2, Il1rap, Il1rn, Il4ra, Il6, Acod1, Itgam, Itgb2, Loxl3, Anxa1, Lrp1, Cd180, Ly86, Lyn, Abcc1, Mmp8, Myd88, Naip2, Naip5, Ncf1, Slc11a1, P2rx7, Per1, Pla2g2d, Pla2g4a, Pla2g5, Serpine1, Ccl21a, Ppara, Ptafr, Ptgs2, Sirpa, Rasgrp1, Ripk1, Rora, Rps19, S100a8, S100a9, Saa3, Ccl12, Ccl2, Ccl22, Ccl3, Ccl6, Ccl7, Ccl9, Cxcl2, Cxcl5, Sele, Selp, Serpina3n, Syk, Sdc1, Tgfb1, Tgm2, Thbs1, Timp1, Tlr1, Tlr4, Tlr6, Tnf, Tnfaip6, Tnfrsf1b, Tyrobp, Mgll, Nt5e, Tlr2, Axl, Map2k3, Tcirg1, Pik3cg, Pbk, Stk39, Ccrl2, Il36a, Cxcl13, Clec7a, Clcf1, Pf4, Stap1, Ppbp, Cd200r1, Cysltr1, Nampt, Serpinb1a, Rtn4, Tnfaip8l2, Prcp, Sting1, Il33, Nfkbiz, Cxcr6, Siglece, Trem2, Pik3ap1, Cd163, Stard7, Chil4, Pld4, Bcr, Tlr7, Havcr2, Stab1, Metrnl, Mylk3, Sbno2, Appl2, Nlrp3, Nr1d1, Nrros, Themis2, Nppa, Adamts12, Cers6, Tarm1, Tlr13, Cd59b, Nr1d2 |

## Supplementary Table 4

**Supplementary Table 4.** KEGG pathway analysis of the 176 DEGs enriched in GO:0006954 process (here only the top 20 significant pathways are listed).

| ID | Category | Description | Count | % | Pathway maps |
| --- | --- | --- | --- | --- | --- |
| mmu04060 | KEGG pathway | Cytokine-cytokine receptor interaction | 35 | 11.99 | Environmental information processing (Signaling molecules and interaction) |
| mmu04062 | KEGG pathway | Chemokine signaling pathway | 25 | 13.02 | Organismal systems (Immune system) |
| mmu04061 | KEGG pathway | Viral protein interaction with cytokine and cytokine receptor | 23 | 24.21 | Environmental information processing (Signaling molecules and interaction) |
| mmu05171 | KEGG pathway | Coronavirus disease-COVID-19 | 22 | 8.91 | Human diseases (Infectious disease: viral) |
| mmu05152 | KEGG pathway | Tuberculosis | 21 | 11.67 | Human diseases (Infectious disease: bacterial) |
| mmu05417 | KEGG pathway | Lipid and atherosclerosis | 21 | 9.72 | Human diseases (Cardiovascular disease) |
| mmu04621 | KEGG pathway | NOD-like receptor signaling pathway | 19 | 9.00 | Organismal systems (Immune system) |
| mmu01100 | KEGG pathway | Metabolic pathways | 19 | 1.21 | Metabolism (Global and overview maps) |
| mmu04613 | KEGG pathway | Neutrophil extracellular trap formation | 17 | 8.29 | Organismal systems (Immune system) |
| mmu05144 | KEGG pathway | Malaria | 16 | 28.07 | Human diseases (Infectious disease: parasitic) |
| mmu04145 | KEGG pathway | Phagosome | 16 | 8.79 | Cellular process (Transport and catabolism) |
| mmu05132 | KEGG pathway | Salmonella infection | 16 | 6.32 | Human diseases (Infectious disease: bacterial) |
| mmu04380 | KEGG pathway | Osteoclast differentiation | 16 | 12.5 | Organismal systems (Development and regeneration) |
| mmu05134 | KEGG pathway | Legionellosis | 15 | 24.59 | Human diseases (Infectious disease: bacterial) |
| mmu05323 | KEGG pathway | Rheumatoid arthritis | 15 | 17.24 | Human diseases (Immune disease) |
| mmu05200 | KEGG pathway | Pathways in cancer | 15 | 2.76 | Human diseases (Cancer: overview) |
| mmu04640 | KEGG pathway | Hematopoietic cell lineage | 15 | 15.96 | Organismal systems (Immune system) |
| mmu04657 | KEGG pathway | IL-17 signaling pathway | 14 | 15.38 | Organismal systems (Immune system) |
| mmu05142 | KEGG pathway | Chagas disease | 14 | 13.59 | Human diseases (Infectious disease: parasitic) |
| mmu04668 | KEGG pathway | TNF signaling pathway | 14 | 12.39 | Environmental information processing (Signal transduction) |
